# Visits to Private Asylums

**Published:** 1902-12-20

**Authors:** 


### VISITS TO PRIVATE ASYLUMS.

By Our Rambling Reporter.

### INTRODUCTORY.

For many years past we have, from time to time, devoted considerable space to a series of notices on the public asylums of Great Britain and Ireland; and we now purpose following this series up by short articles on our private asylums. In all cases these will be written after special visits have been made to the institutions.

It happens now and then that the daily press inserts sensational paragraphs about these private asylums, and the general public, knowing little or nothing about the status or administration of such institutions, are prone to believe these adverse comments when they first appear, and pay but small attention to the real facts of the case, or to the refutations or explanations which are, practically, invariably forthcoming.

Whether the very existence of these private asylums is a thing which can be theoretically justified or not, is a point on which we need not do more than lightly touch. It might be argued, very plausibly, that no one detained anywhere against his will, be it even in a palace, should be so detained except by State-paid officials acting in accordance with the law; and, speaking merely from the theoretical standpoint, we might feel disposed to admit the force of this argument, which would be also applicable to the lunatic hospitals as these are not managed by State-paid officials any more than are private asylums. But these private asylums exist, and they are likely to exist for a great many generations yet to come, and they so exist because good work is being done by them, and because they supply a want felt by a large section of the population unfortunate enough to have insane relatives. Many of these people would shudder at the very idea of placing their friends in any kind of public asylum, and we can sympathise with this feeling.

The safeguards against abuse by these private asylums are sufficiently great to prevent it, but as a matter of fact no proprietor or licensee wishes to take any advantage. It would be as much against his interest as it would be against his principles. Let us, however, look at some of these safeguards. Excepting in the case of emergency orders which are valid for a few days only, or in the case of voluntary boarders, no patient can be detained in any private asylum unless two medical men certify to the insanity, and unless such certificates are accompanied by a magistrate's order. Within a few days of the reception of the patient, copies of these papers must be sent to the office of the Commissioners in Lunacy. The asylum is visited twice a year by the Commissioners, and four times a year by a committee of county magistrates who are accompanied by a medical practitioner appointed by themselves, and when the asylum is not large enough to require a resident medical officer, a local practitioner regularly attends the patients. To improperly admit anyone to a private asylum and detain him in it would therefore imply the roguery or incapacity of at least two Commissioners in Lunacy, of several county magistrates, of two or more medical men, and of the proprietor of the asylum—a combination or a conspiracy that could hardly occur in this country.

There are in England about 68 of these private asylums or licensed houses as they should be called; and the accommodation provided in these houses ranges from three beds up to 670. The last Lunacy Act contained one singular provision concerning them. No new private asylum was to be licensed, nor could any existing private asylum have its numbers increased beyond those it was licensed for on the passing of the Act. The object of this clause is most difficult to arrive at. It is perhaps wholly incomprehensible, as the Commissioners in Lunacy who grant licenses to the Metropolitan asylums and the County Magistrates who grant those for the provincial ones always had it in their power to refuse the renewal or the increase of the license.

The population of the country is an ever-increasing one, and the proportion of the insane would seem to keep pace with it, if it do not increase beyond the proportion of former years. It is a fact, too, that the lunatic hospitals which were at first provided for the care of those who could not maintain themselves in private asylums, but who were above the rank of paupers—it is a fact that these hospitals which were intended by their founders to be veritable charitable institutions, have become, or at least many of them have become, institutions in which their charitable nature has been left in the background, and they now receive patients at as high rates of board as do the real private asylums. This departure has made the lot of many of the poorer insane and the friends of the insane a grievous one. The patients have been sent to our county asylums to associate with paupers, or their friends have had to pinch themselves to pay the payments in private asylums. It is consistent with our knowledge that the proprietors of private asylums have in some cases held out a helping hand to these afflicted people and have admitted patients far below their usual rates, thus saving the feelings of the patient and his friends.

The management of these lunatic hospitals is, in most cases, admirable; but that is no justification for the position they have taken up, and it should be added that some of them still act up to the intention of their founders, and are really charities. On this subject the Commissioners in Lunacy have the following very sensible remarks: "Few patients are received into hospitals at low or moderate rates. Accommodation of a very good kind can be, and is, provided in many licensed houses for 30s. weekly, and we regret that more provision for the middle class is not made at that figure, or still lower, by institutions originally founded as charities for the insane. . . . Some are making large profits, but these profits appear too often to be expended, not in the extension of provision for cases only able to meet moderate payments, but in accommodation calculated to attract the wealthier class who are not in the same strait for suitable asylum accommodation, care and treatment." Every word of that paragraph is as true now as when it was written; but what steps the Commissioners have taken to prevent the admission of rich patients into these hospitals, or to increase the admission of deserving suitable cases we do not know.

While it would be most undesirable to increase the number of the patients in the larger of our private asylums, it is indisputable that many of the smaller ones would be improved by additions to the number permitted by their present license; and it is not too much to hope that Parliament will, at no distant date, see fit to allow these additions, as thereby better classification of the patients could be made and lower rates charged for their maintenance. But there ought always to be a considerable number of very small asylums in which the patients can live like members of one family, and this is more especially the case where only ladies are received and where somewhat high rates of board are charged.

On the 1st of January, 1902, the total number of registered private patients in England was 9,135. Of this number, 1,824 were in county asylums; 3,702 in lunatic hospitals, 254 in Naval and Military hospitals, 464 were single patients, and 2,891 were in private asylums. We believe that the number of single patients might be enormously increased as there must be many more than one private patient in 20 suitable for treatment in private houses; and it is further our opinion that the lunatic hospitals should revert to their original charitable objects and should practically receive only those who are incapable of paying more than a part of their maintenance.

Some of our county asylums have exercised the powers conferred on them by the Lunacy Act, and have provided accommodation for private patients. The cases received are for the most part those who cannot afford more than 20s. a week, and they are often received at much less. The extension of this system will have two important results. Firstly, it will prevent the admission to our county asylums as paupers of those able to pay small sums, and whom the lunatic hospitals have declined to take, and secondly, it will further reduce the charges which the country has to pay for its pauper lunatics, because profits will accrue from the reception of the private patients, which profits can be applied to reduce some of the charges of the County Rate.

The purchase by the State of all the private asylums is no longer heard of, nor, indeed, was it ever within reasonable distance of being carried out. The directions in which changes would seem desirable are: the extension of a certain number of our private asylums, the practical restriction of lunatic hospitals to receive only charitable cases; and the provision at nearly all our county asylums for the class just above the so-called pauper lunatics.

